# Processing of ingredients and diets and effects on nutritional value for pigs

**DOI:** 10.1186/s40104-017-0177-1

**Published:** 2017-06-01

**Authors:** Oscar Javier Rojas, Hans Henrik Stein

**Affiliations:** 1 Present Address: Devenish Nutrition, Fairmont, MN 56031 USA; 2 Department of Animal Sciences, University of Illinois, Urbana, IL 61801 USA

**Keywords:** Chemical treatments, Enzymes, Particle size, Pig, Processing, Starch

## Abstract

A conventional diet based on corn and soybean meal fed to pigs is usually provided in a mash form and in most cases, processing other than grinding and mixing is not used. However, due to the high cost of energy in pig diets, use of high fiber ingredients such as soybean hulls, distillers dried grains with solubles, and wheat middlings has increased. High fiber concentrations in the diet usually results in reduced energy and nutrient digestibility due to the low capacity of pigs to digest fiber, which negatively impacts growth performance and carcass composition of the pigs. Feed processing technologies such as changes in grinding procedures, expansion, extrusion, pelleting, use of enzymes or chemical treatments may, however, be used to solubilize some of the cellulose and hemicellulose fractions that form the cell wall of plants in the ingredients, and therefore, increase nutrient availability. This may have a positive effect on energy digestibility, and therefore, also on pig growth performance and carcass composition, but effects of different feed technologies on the nutritional value of feed ingredients and diets fed to pigs are not fully understood. It has however, been demonstrated that reduced particle size of cereal grains usually results in increased digestibility of energy, primarily due to increased digestibility of starch. Extrusion or expansion of ingredients or diets may also increase energy digestibility and it appears that the increase is greater in high fiber diets than in diets with lower concentrations of fiber. Chemical treatments have not consistently improved energy or nutrient digestibility, but a number of different enzymes may be used to increase the digestibility of phosphorus, calcium, or energy. Thus, there are several opportunities for using feed technology to improve the nutritional value of diets fed to pigs.

## Background

The global production of processed feed exceeds one billion metric tons per year. Thus, a large part of the feed that is fed to livestock is processed in one form or another. In addition, even if complete feed is not produced, many feed ingredients are processed using one or more feed processing techniques before feed is consumed. As a consequence, any impact of feed processing on the nutritional value of the feed is important to take into consideration. Most ingredients are ground before being consumed, which reduces the particle size and increases digestibility [1]. Feed ingredients are also sometimes heated, which may reduce concentrations of antinutritional factors, but effects of heating on energy and nutrient digestibility have not been consistent and may be both negative and positive [2]. Other processing techniques that may be used include expander processing [3, 4], pelleting [5, 6], and extrusion [3, 7]. The nutritional value of feed ingredients or diets may also be improved by chemical or enzyme treatments because such treatments may solubilize the cellulose and hemicellulose fractions that form the cell wall of plants. Some of the chemicals used to increase fiber digestibility are sodium hydroxide (NaOH) [8–10], ammonium [11, 12], calcium oxide [13], and calcium hydroxide (Ca(OH)_2_) [14]. Benefits of microbial phytase in terms of increasing the digestibility of phosphorus [15, 16] and calcium [17, 18] are well documented, but cellulase, hemicellulase, xylanase, β-glucanase, α-galactosidase, or carbohydrase mixtures may potentially be used to increase energy and fiber digestibility in feed ingredients and diets [19–24]. There is, however, a lack of information about effects of many of these processing techniques on energy and nutrient digestibility and the utilization of feed by pigs. There is also a lack of information about how combinations of different processing techniques may impact feed utilization by pigs. It is, therefore, the objective of this review to discuss processing techniques and enzyme additions that may improve the nutritional value of feed ingredients or diets fed to pigs. We have included available information obtained with pigs where available, but on a few occasions, no information has been published and in those cases we have extrapolated data from other species.

## Particle size of feed ingredients

### Measuring particle size

Determining the mean particle size of feedstuffs that are commonly used in diets fed to pigs is not a well-established practice in feed mills. However, energy and nutrient digestibility may be increased as the particle size of feedstuffs decreases [1, 25–29]. Therefore, it is important to determine the optimal particle size of feed ingredients to maximize energy and nutrient digestibility.

The American Society of Agricultural Engineers has published a procedure for determining particle size and calculating the fineness of feedstuffs [30]. Particle size distribution and mean particle size of feedstuffs are determined using 100 g of feedstuff that is placed on the top of a stack of test sieves (i.e., U.S sieve # 4, 6, 8, 12, 16, 20, 30, 40, 50, 70, 100, 140, 200, 270, and a solid metal pan), which are stacked from the biggest to the smallest aperture size sieve. The test sieves are located in a vibratory sieve shaker for 10 min. The amount of feedstuff that is accumulated in each of the test sieves is recorded and weighed to calculate particle size distribution and mean particle size. After determination of particle size, the surface area is calculated using the mean particle size of the feedstuff as a reference [30].

### Mills used for grinding

Grinding is used to reduce the particle size of a feed ingredient and it is accomplished with the use of different types of mills. The most common mills used in the industry are roller mills and hammer mills. Ingredients such as distillers dried grain with solubles (DDGS) and soybean meal (SBM) are often ground during the production process, and in most cases, no further grinding is needed for these ingredients before diets are mixed. In contrast, cereal grains and pulse crops are usually not ground prior to entering the feed mill, and these ingredients, therefore, need to be ground.

In the feed industry, there are different preferences for use of roller mills or hammer mills. These preferences are often based on the grinding capacity needed, electricity efficiency and types of feedstuffs used [5]. However, roller mills require more oversight and they are more complicated to operate and manage than hammer mills, but they have improved energy efficiency, and provide a more uniform particle size compared with hammer mills. Thus, there is less variation among the size of particles if a roller mill is used compared with a hammer mill [5, 31]. Hammer mills increase losses of moisture from the grain, are more noisy, and are more costly to maintain than roller mills [32], but a hammer mill system can be installed for about 50% of the cost of a roller mill [33]. Corn that is ground with a roller mill compared with a hammer mill contains more uniform edges and the shape of the particles tends to be more spherical [34]. However, a more uniform particle size distribution is also observed with the same mean particle size if a roller mill rather than a hammer mill is used, which results in a greater digestibility of dry matter (DM), gross energy (GE), and N, but this does not affect growth performance [31]. Roller mills may be stacked so the grain is rolled not only once, but 2, 3, or even 4 times [35]. This procedure allows roller mills to produce an end-product with a particle size of less than 500 μm.

Historically, most feed mills have used either roller mills or hammer mills, but not both, but recently, advances in milling technology have introduced systems where ingredients are first rolled using 1, 2, or 3 sets of rollers and then processed in a hammer mill. This technology is known as “multiple stage grinding”, and it is believed that this results in a more uniform particle size and reduced cost of grinding, but no comparative data between multiple stage grinding and single stage grinding have been reported. It is also possible to sieve the material after the rollers so that only the larger particles are guided to the hammer mill, whereas smaller particles by-pass the hammer mill. Use of this procedure will minimize electricity usage and results in the most uniform particle size.

Electricity used to process feedstuffs is an important component in a feed mill’s budget. Corn milled in a hammer mill at 600 μm rather than 1,000 μm increased energy usage, and when particle size was decreased from 1,000 to 400 μm, the energy usage increased almost 2.5 times [1]. The production rate (ton/h) also decreases as particle size is reduced [36]. Likewise, electricity costs are more expensive for hammer mills compared with roller mills [33]. Energy usage is also affected by the type of cereal that is ground [5].

### Effect of particle size on digestibility of energy and nutrients in cereal grains

Research to identify an optimal particle size of cereals has been conducted and effects of particle size on energy and nutrient digestibility have been reported [1, 25–29, 31, 37–39]. Most recommendations for optimal particle size were generated between the 1960s and the 1990s depending on the type of cereal grain, type of milling, and physiological state of the pig (e.g., weanling pig, growing pig, finishing pig, or sow). However, in most cases, a reduction in the mean particle size to a range between 485 to 600 μm had a positive effect on nutrient and energy digestibility and growth performance [29, 31].

A reduction of particle size of wheat from 920 to 580 μm increased apparent total tract digestibility (ATTD) of starch, but not of GE [40]. However, pigs fed a barley-field pea diet with a particle size of 400 μm had an increase in ATTD of GE, DM, crude protein (CP), and GE compared with pigs fed the same diet ground to 700 μm [41]. A linear increase in ATTD of GE and CP and in the standardized ileal digestibility (SID) of amino acids (AA) also has been observed when particle size of lupins was reduced from 1,304 to 567 μm [42]. Likewise, reduction of the mean particle size of field peas results in an increase in the digestibility of starch and energy and therefore also in an increase in DE [43]. The ATTD of DM and GE, and the concentration of metabolizable energy (ME), increased when pigs were fed DDGS ground to 308 μm compared with pigs fed DDGS ground to 818 μm, but particle size did not affect the ATTD of N and P [44]. Reduction of particle size of corn from 500 to 332 μm also increased the rate of phytate degradation [45], but generally, particle size of corn grain or corn DDGS does not affect P digestibility [29, 44].

Several experiments have focused on evaluating corn particle size because corn is one of the most common ingredients used in pig diets. The ATTD of DM, N, and GE in corn increased 5, 7, and 7 percentage units, respectively, when particle size was reduced from 1,200 to 400 μm [38], but the type of mill used to grind the corn may have an effect on energy and nutrient digestibility [31]. A reduction of the mean particle size of corn from 865 to 339 μm linearly increased the apparent ileal digestibility of starch and GE and the concentration of DE and ME, but this was not the case for the standardized total tract digestibility of P or the SID of AA [29]. Similar results were observed by Giesemann et al. [46], who reported that finishing pigs fed a corn-based diet with a particle size of 641 μm had an increased ATTD of DM, N, and GE compared with pigs fed a diet with a mean particle size of 1,500 μm. Likewise, a reduction in particle size from 900 to 300 μm in corn and sorghum improved the ATTD of GE [36, 47], and an improved ATTD of GE and ether extract was observed as the particle size of corn grain was reduced [48]. The use of pelleting in combination with grinding also may increase ATTD of DM, GE, and N [1]. In a 24 d experiment, energy and DM digestibility was determined on d 9 and pigs fed corn ground to 1,000 μm had a reduced ATTD of DM and GE compared with pigs fed corn ground to 500 μm [27]. The ATTD of GE, DM, and N also improved as the particle size of corn was reduced from 1,200 to 400 μm [25]. However, particle size did not affect urine N excretion, but as particle size of corn was reduced from 1,200 to 400 μm, the ME of corn increased from 3,399 to 3,745 kcal/kg [25].

The ATTD of GE also is improved linearly if particle size of sorghum is reduced [36]. A reduction of particle size of SBM from 949 to 185 μm had no effect on average SID of indispensable AA or dispensable AA, but a linear increase in the SID of isoleucine, methionine, phenylalanine, and valine was observed as particle size was reduced [28]. However, energy digestibility of SBM was not affected by decreasing the particle size of SBM from 949 to 185 μm. Nevertheless, it was suggested that SBM ground to 600 μm will have the best AA and energy digestibility [28].

The ATTD of N and DM in wheat also increase as particle size is reduced from 1,300 to 600 μm [26], but this is not the case for barley because the ATTD of organic matter (OM), energy, or CP is not affected by particle size [49]. This indicates that the effect of reduction in particle size is unique and depends on each specific ingredient.

The reason for the increased DE and ME that have been observed in most experiments as the particle size has been reduced is most likely an increased ileal digestibility of starch and therefore also an increased ileal digestibility of energy [29]. However, in some cases, increased digestibility has been observed if ingredients that have high concentrations of fiber, but do not contain starch have been used [44] and it is, therefore, possible that in some ingredients, the fiber matrix may capsulate energy containing nutrients such as starch, lipids, and protein. In such cases, the finer grinding may disrupt the fiber matrix and therefore make energy containing nutrients accessible to digestive enzymes. To further understand the mechanisms that are involved in improving energy digestibility of different feed ingredients, research needs to be directed at determining the mechanism for energy improvement as the particle size is reduced. This type of research should be conducted in starch containing as well as non-starch containing ingredients.

### Effect of particle size on ulcer development

The stomach of the pig has 4 different regions (esophageal region, cardiac region, fundic region, and pyloric region) [50]. The esophageal region is the non-glandular region, whereas, the cardiac, fundic, and pyloric regions are the glandular regions. Each region has specific characteristics to maintain the function of the stomach, but the functions of the stomach may be interrupted if pigs develop ulcers, and it is possible that particle size of feed ingredients impact the risk of pigs developing ulcers. The esophageal region is the region that is most at risk of developing gastric ulcers if pigs are fed ingredients with a reduced particle size [51–54] because the mucus in the glandular portion of the stomach has a protective function [55, 56]. However, a reduced particle size of grain is not the only factor that may trigger development of ulcers. There are other factors such as type or intensity of production [57, 58] and type of housing [59] that also may increase the risk of pigs developing ulcers. The development of ulcers increases as pigs are fed pelleted diets that contain corn ground to 400 μm compared with pigs fed non-pelleted diets [1, 31]. However, growth performance may not always be affected by the presence of ulcers, and pigs fed pelleted diets usually have greater average daily gain and G:F than pigs fed un-pelleted diets.

Development of ulcers is considered one of the major economical losses in the swine industry [60] and the presence of esophagogastric ulcers have increased in the U.S. pork industry due to increased use of pelleting [5]. In the UK, 79% of pigs from 60 farms had some level of ulcers [61] and in a survey related to the presence of gastric ulcers in pigs on 16 commercial farms in the UK, it was observed that 19.1% of the commercial farms had some prevalence of ulcers [59]. It is hypothesized that the formation of ulcers starts within 7 d after pigs are provided a diet ground to a small particle size and it is also assumed that keratinization and erosions of stomach tissue may be ameliorated when pigs are fed a coarse diet for 7 d [54]. It has also been proposed that this may be achieved if pigs are fed coarse diets 40 h prior to slaughter [52]. Development of ulcers is followed by colonization of *Helicobacter* spp. and the presence of this microorganism is more evident in the fundic and pyloric regions than in the esophageal and cardiac regions [62]. Pigs fed either a finely ground diet or a pelleted diet have a greater secretion of chloride in the stomach compared with pigs fed a coarsely diet or a non-pelleted diet [63], which promote the presence of *Helicobacter* spp. in the stomach [64, 65] and reduces pH.

Pepsin activity is also increased as particle size of corn decreases [54]. Because o fthe increase in chloride secretion, pigs fed diets that are either finely ground or pelleted have greater concentrations of chloride in the esophageal region of the stomach compared with pigs fed either coarse or unpelleted diets [63]. This may be due to an increased mixing in the stomach and more watery digesta, which also results in an increase in HCL secretion.

Pigs fed corn with less variation in particle size tend to have less keratinization in the stomach [31] and pigs fed corn ground to 400 μm have more ulcers and keratinization in the esophageal region compared with pigs fed corn ground to 1,200 μm [1]. A linear increase in the severity of parakeratosis in the esophageal region of the stomach in finishing pigs was observed as particle size of corn was reduced from 865 to 339 μm, but this change in keratinization did not impact pig growth performance [66]. Pigs fed diets containing wheat ground to 600 μm also developed more ulcers and had more tissue keratinization compared with pigs fed diets containing wheat ground to 1,300 μm, but this did not have an effect on G:F [26]. Likewise, when sows were fed corn ground to 1,200 μm, only 25% of the sows developed ulcers, but if sows were fed corn ground to 400 μm, 77% of the sows developed ulcers [1].

Whereas the mechanisms for ulcer developments are established as pointed out above, research is needed to determine strategies for prevention of ulcers. It is possible that there are differences among different genetic lines of pigs in their susceptibility to ulcers, but research to demonstrate this has not been conducted. It is also possible that dietary fiber reduces the risk of pigs developing ulcers and that the particle size of high fiber diets can be reduced compared with low fiber diets without increasing the risk of pigs developing ulcers but this has also not been experimentally verified.

### Effect of particle size on pig growth performance and sow productivity

Reduction of cereal grain particle size may increase enzyme surface action, which leads to increased energy and nutrient digestibility [27–29]. However, this increase in digestibility is not always translated into a positive effect on growth performance because pigs may compensate for a low digestibility by eating more feed. The best results on growth performance are obtained in weanling pigs and finishing pigs if wheat is ground to 600 and 1,300 μm, respectively [26].

Feed intake may be improved if particle size of wheat is reduced from 1,200 to 980 μm, but this did not have an effect on overall gain to feed (G:F) [67]. Likewise, pigs (93 to114 kg) fed wheat that was ground to 600 μm had improved G:F compared with pigs fed wheat ground to 1,300 μm [26], but the same effect was not observed from 67 to 93 kg. In contrast, Hancock, Behnke [5] reported that for each 100 micron decrease in particle size of corn, the G:F ratio in growing pigs will be improved by 1.3%, and Wondra et al. [31] reported that the G:F ratio was 7% greater in pigs fed corn ground to 400 μm compared with pigs fed corn ground to 800 μm. These observations are in agreement with data reported by Amaral et al. [37] and Rojas et al. [66]. Pigs have a greater preference for corn when the particle size is reduced than for sorghum that was ground to the same particle size as corn [36] and Kim et al. [40] hypothesized that reduction of particle size does not have the same effect among all cereals grains. Pigs fed corn ground to 400 rather than 1,000 μm had reduced average daily feed intake and increased G:F, which is likely a result of the greater energy value in corn ground to 400 μm compared with corn ground to 1,000 μm [1]. Similar results were reported by Paulk et al. [68] who observed that finishing pigs fed diets containing sorghum ground to 319 μm improved G:F compared with pigs fed a diet containing sorghum ground to a mean particle size of 724 μm. In contrast, growth performance of pigs fed SBM that was ground to 639 μm or 444 μm was not different from that of pigs fed SBM ground to 965 or 1,226 μm [39]. It was hypothesized that the reason for this observation is the low inclusion level of SBM in the diet [39]. Thus, the effect of reduced particle size may only be measurable if a high inclusion rate of the ingredient is used in the diet.

Carcass dressing percentage was increased linearly in pigs fed corn ground from 865 to 339 μm [66] and there was a tendency for a greater carcass dressing percentage when pigs were fed a diet containing sorghum ground to 319 μm compared with pigs fed a diet containing sorghum ground to 724 μm [68]. Likewise, pigs fed corn ground to 400 μm compared with pigs fed corn ground to 1,000 μm had an increase in carcass dressing percentage [1]. The reason for this observation may be that the weight of the intestines is reduced with reduced particle size of corn [66]. In contrast, Mavromichalis et al. [26] reported that there was no effect on carcass dressing percentage when pigs were fed wheat ground to 600 μm compared with pigs fed wheat ground to 1,300 μm. Thus, it appears that the positive effect of smaller particle size on dressing percentage that is observed for corn and sorghum, may not always be seen for other cereal grains and more research to investigate effects of particle size on dressing percentage of pigs fed wheat, barley, triticale, and rye is needed.

Relatively little research investigating the effect of particle size on sow BW and litter performance has been reported. A reduction in particle size of corn from 1,200 to 400 μm does not affect BW or back fat losses in lactating sow [38]. However, there was a linear decrease in ADFI of sows as particle size of corn was increased from 400 to 1,200 μm and a decrease in litter BW gain was also observed [38].

### Effect of particle size on feed flowability and handling

There is relatively little information about the effect of particle size on feed flowability and handling, but it has been hypothesized that reduced particle size may result in poor flowability [69]. This concurs with observations indicating that flowability of diets is reduced as particle size of DDGS or corn is reduced [44, 66]. Likewise, SBM ground to 639 μm had a greater angle of repose than SBM ground to 965 μm [39], but if a bowl type feeder is used and if feed is added twice daily, a reduced particle size does not reduce flowability of the diet [31]. Likewise, pelleting of diets will also prevent the bridging problems, dustiness, ingredient segregation, and increased bulk density that are common problems when diets are ground to less than 600 μm [1, 70]. The palatability of corn with a particle size of 444 μm was not less than that of corn ground to 619 μm when fed to lactating sows [71], which indicates that dustiness may not have been a problem for the sows eating the feed with the lower particle size.

## Thermal treatments

Effects of feed processing techniques on energy and nutrient digestibility of diets or feed ingredients have been investigated [7, 72–76] and it is generally reported that the nutritional value of ingredients and diets may be improved by feed processing [77]. Feed processing often involves application of a source of heat, but excessive heat may result in the Maillard reaction [78]. The Maillard reaction takes place between an amino group in an AA and a carbonyl group of a reducing sugar [79], which reduces the availability and digestibility of AA [80–82]. Heating followed by cooling may also result in retrogradation of the starch, which will then become less digestible and, therefore, the energy value may be reduced [83]. Thermal treatment of diets may also reduce the efficiency of phytase and other exogenous enzymes because of the heat that is applied [84] and phytase and other enzymes need to be thermostable or be sprayed on the pellets after production.

### Steam conditioning

The main objective of steam conditioning is to establish conditions that will result in production of a durable pellet [5]. During conditioning, the temperature increases to 75 °C and the moisture increases 3 to 4 percentage units in the feed mixture for approximately 1 min [85]. Addition of either steam or water results in formation of a liquid layer on top of the ingredients that helps bind certain particles in the mixture [86]. It is believed that the durability of the pellet is increased and the heat damage of the starch is reduced if ingredients are conditioned prior to pelleting [77]. Therefore, the conditioning step is important because there are several factors that may impact the quality of the pellet such as particle size of ingredients and the type of ingredients included in the diet [5].

Steam conditioning may be completed by a single pass or a 2-pass conditioner, which influences the length of time the ingredients will be in the conditioner. The longer feedstuffs are exposed to the steam and the greater the temperature is, the greater is the starch gelatinization and protein denaturation [5, 87], because the starch granules become hydrated and swell due to absorption of water [88]. However, rapid cooling after heating may lead to formation of retrograded starch, which leads to formation of crystals that reduce enzymatic starch digestibility [89, 90] and cooling needs, therefore, to be controlled to reduce the risk of creating retrograded starch. There is, however, no data to demonstrate the exact conditions that are needed for optimizing effects of conditioning and the subsequent cooling process and more research in this area is clearly needed.

### Pelleting

Use of steam and pressure are the principles behind the pelleting technology. Steam increases the temperature of the feed and the steamed ingredients are subsequently pelleted to a determined pellet size using pressure [77]. Effects of different pellet sizes have been investigated, but it has been suggested that diets for nursery and finishing pigs may be processed using a single die with 4 to 5 mm holes without affecting growth performance [5]. Effects of die thickness on the SID of AA in corn and wheat fed to pigs was investigated, but no significant effects were observed when the size of the die increased from 16 to 24 mm or from 16 to 20 mm [91]. Pellet hardness and pellet durability index are acceptable indicators of pellet quality [92] and it is believed that expansion or extrusion prior to pelleting may increase the pellet durability index in diets based on cereal grains [93]. Pelleting changes the physico-chemical characteristics of the ingredients due to the heat that is applied during the process [77], and pelleting usually improves feed intake of weanling pigs compared with diets provided in a mash form [94].

Starch in cereals grains that are pelleted is more likely to be digested in the small intestine due to the gelatinization of starch that may be accomplished by pelleting [95]. Likewise, a decrease in dustiness and increased handling properties, bulk density, and reduction in segregation of components in feed ingredients are some of the advantages of using pelleting [5, 85]. However, the acquisition and maintenance of equipment for pelleting may be expensive [85].

Pelleting a corn-soybean meal diet increased digestibilities of DM, N, and GE by 5 to 8% compared with feeding the same diet in a meal form [1]. This concurs with observations by Rojas et al. [76] who reported that pelleting different types of diets regardless of the level of fiber [i.e., 7, 11, or 20% neutral detergent fiber (NDF)] improved the apparent ileal digestibility of GE, DM, and most indispensable AA and the ATTD of GE compared with un-pelleted diets. Likewise, Lahaye et al. [96] reported that pelleting a wheat-canola meal diet improved ileal digestibility of CP and AA and this is also the case if field peas are pelleted [7], and similar results for diets containing wheat and SBM were reported [97]. Diets fed to growing and finishing pigs based on corn and wheat middlings that were pelleted increased the digestibility of GE and G:F [98]. It is possible that the reason for the increase in GE is that under certain circumstances, the ATTD of ether extract is increased if diets are pelleted [99]. However, due to significant microbial synthesis of fat in the hindgut of pigs, data for the ATTD of fat are not representative of lipid absorption and these results should, therefore, be viewed with caution [100]. In addition, due to formation of Ca-lipid soaps in the intestinal tract of pigs, determination of ATTD values for ether extract without prior acid hydrolysis is likely to yield inaccurate results, which further makes published data for effects of pelleting on the ATTD of fat difficult to interpret. It has, however, been demonstrated that intact fat in plant ingredients is less digestible than extracted fat that is added to diets [100, 101] and it is, therefore, possible that conditioning or pelleting may disrupt some of the matrixes that prevents fat in intact ingredients from being digested if no conditioning or pelleting takes place. However, only limited research has been directed at determining effects of pelleting on the ileal digestibility of acid hydrolyzed ether extract, but small improvements have been observed [101, 102]. However, there is a need for more research in this area.

The repeated observations that pelleting results in increased ileal digestibility of both starch and AA indicate that pelleting, or possibly the conditioning that takes place before pelleting, may increase starch gelatinization and change protein confirmation, which in turn increases the ability of digestive enzymes to digest starch and protein. However, the observations that pelleting also results in increased total tract digestibility of fiber indicates that pelleting possibly also increases the solubility of fiber, which then results in increased fermentation in the hindgut of the pig.

Pelleting often results in an increase in feed conversion by 4 to 12 percentage units [94, 103–106], and ADG is also often increased by pigs fed pelleted diets compared with pigs fed diets in a meal form [104, 107–109]. The main reason for these observations is that feed wastage is reduced and digestibility of energy is improved because of gelatinization of starch [110, 111] that occurs when cereal grains are processed in the presence of heat. Therefore, pelleting may also impact feed intake and gut function of the pig [85]. Recently, it was reported that pigs fed pelleted diets had a greater feed efficiency compared with pigs fed meal diets, and the reduced growth performance of pigs that was the result of feeding diets containing high-fiber by-products was ameliorated if the diet was pelleted [112]. This observation concurs with recent data demonstrating that the ME of diets is increased by pelleting [76].

### Extrusion

In association with pelleting, extrusion is a technology that is often used in the feed industry. In the United States, only 5% of pet feed is not extruded [113], which demonstrates the importance of this technology for the pet feed industry. The extrusion process consists of pressuring the feed material through a barrel by the use of single or twin-screw extruders, which results in generation of heat [5, 88, 111]. Both types of extruders may be used on the whole diet or on individual ingredients. The objective of extrusion is to increase energy and nutrient digestibility in cereal grains, which is expected to have a positive effect on feed conversion rate and possibly growth performance of pigs [5]. Extrusion results in a more severe change in the physico-chemical characteristics of the feedstuff compared with pelleting [77] because of the change in temperature, pressure, friction, and attrition of the feedstuffs inside the extruder [5]. Extrusion of the entire diet compared with pelleting improved feed conversion by 8% and DM and CP digestibility by 3 and 6%, respectively [114]. However, feed intake of pigs is not always improved when diets containing wheat or sorghum are extruded [115]. Ileal digestibility of DM is improved by extrusion of corn, but AA digestibility is not different between extruded and non-extruded corn [116]. However, the ileal digestibility of CP may be greater in extruded soybean meal compared with non-extruded soybean meal [117], but that is not always the case [118]. Extrusion of field peas has a positive effect on the ATTD of GE and on the apparent ileal digestibility of most indispensable AA [7, 119] and GE [116] and the DE of field peas is improved by 4.8% by extrusion [7]. Extrusion or extrusion in combination with pelleting of a diet containing corn, soybean meal, DDGS, and soybean hulls improved the ileal digestibility of GE, starch, DM, CP, and most indispensables AA compared with an un-processed diet [76]. Therefore, there is an opportunity for increasing energy and nutrient digestibility if ingredients that have high concentrations of fiber are extruded, but it may not always be economical to extrude diets for growing-finishing pigs [5]. Apparent total tract digestibility of DM and CP was not different when a flaxseed-field pea mix was extruded using either a twin-screw extruder or a single-screw extruder [119]. However, ATTD of GE and the concentration of DE were greater in the diet extruded using a single-screw compared with the diet extruded using a twin-screw extruder [90]. Likewise, extrusion also may increase the solubility of dietary fiber, which in turn may result in an increased energy digestibility because soluble fibers are much more fermentable by pigs than insoluble fibers [120]. As a result of the positive effects of extrusion on digestibility and feed efficiency, some feed companies in Europe extrude diets for pigs and most of the compound feed in Europe is pelleted. However, the benefits of extrusion are not fully understood and because positive results are not obtained in all situations more research in this area is needed.

### Expansion

Expansion is also known as a shear conditioning process. The reduced temperature and retention time that feed ingredients are exposed to in the expansion process are the main differences between this process and the extrusion technology [121]. This is the reason there is less starch gelatinization if feed ingredients are processed using the expansion technology compared with using the extrusion technology [75]. It has been proposed that pelleting may be replaced by expansion [77], because during expansion, the physico-chemical characteristics of the feed are modified [122] due to the high pressure that is used in the process [5]. However, nutrient and energy digestibility were not improved by pigs fed expanded diets based on wheat and barley compared with pigs fed un-expanded diets [123], but fiber digestibility may be improved by expansion [122]. In contrast, Traylor et al. [93] reported that there was an increase in energy and nutrient digestibility when growing pigs were fed an expanded corn-SBM based diet compared with pigs fed an un-expanded corn-SBM based diet. However, digestibility of DM, NDF, and CP were not improved if pigs were fed an expanded diet containing barley and wheat bran-wheat middlings [124].

It is unusual that expanded feed is offered to pigs in mash form. Instead, most expanded feed also goes through a steam condition step and pelleting [124–126]. The pellet durability index of a corn- and barley-based diet was improved by adding water into the mixer followed by expansion of the diet [126]. It is, thus, possible that expansion in combination with pelleting may result in a better quality pellet [5].

Usually, a complex phase 1 diet contains corn, SBM, soybean oil, and animal protein. Expansion of different portions of a complex diet (e.g., corn, corn-SBM, or corn-soybean meal-oil) in combination with highly digestible animal protein results in an increase in ADG when fed to weanling pigs compared with pigs fed a whole complex diet that was expanded [125]. If a wheat-fish meal-SBM based diet was either expanded or extruded and fed to weanling pigs for 36 d, the greater G:F in pigs fed the extruded diet compared with pigs fed the expanded diet was mainly due to a greater digestibility of starch in the extruded diet [3]. However, Millet et al. [127] reported an increased G:F for pigs fed an expanded diet compared with pigs fed the same diet in meal form, but no difference in ADG was observed, which indicates that expansion may have improved energy digestibility.

## Chemical treatments

Most research using chemical treatments has been conducted using ruminant animals because it is believed that mainly high fiber ingredients will benefit from chemical treatments. However, some high fiber feed ingredients such as DDGS, other corn co-products, and co-products from wheat or rice have become important ingredients in diets fed to pigs due to their relatively low cost [128]. Almost 90% of the total fiber in DDGS is insoluble fiber and only 40% of insoluble fiber in DDGS is fermented [120]. In contrast, more than 90% of the soluble dietary fiber is fermented, but soluble fiber accounts for only 10% of the total fiber in DDGS [120]. Therefore, any treatment that can solubilize some of the insoluble fiber in DDGS or other cereal co-products is expected to result in increased energy contribution from the fibers because of the increased fermentability of soluble fiber.

### Sodium hydroxide

Sodium hydroxide is considered a hydrolytic agent that may solubilize the hemicellulose, lignin, and silica constituents of the plant cell wall. The solubilization is mainly due to changes in the lignin-hemicellulose matrix that takes place when the cell wall is in contact with NaOH [8]. Changes in the plant cell wall may improve access of microbial enzymes to the constituents of the plants [8]. Sodium hydroxide has also been used to remove feathers from hens as an alternative procedure compared with rubber picking fingers, but the nutritional value of the feathers removed with this procedure was not improved compared with the conventional method [129].

There is limited information about the effect of NaOH treatments on energy and nutrient digestibility of ingredients fed to pigs, whereas much research has been conducted with ruminant animals [9, 10, 130–132]. Pigs fed bird-proof sorghum treated with NaOH increased nitrogen and energy digestibility [133]. Likewise, pigs fed *Leucaena leucocephala* leaf meal that was treated with NaOH had improved N retention compared with pigs fed untreated *Leucaena leucocephala* meal [134], which may be due to a reduction in the concentration of tannins in *Leucaena leucocephala* leaf meal treated with NaOH [135]. However, pigs fed cooked soybeans that were treated with NaOH had reduced growth performance compared with pigs fed untreated cooked soybeans [136], which may have been caused by reduced palatability of the diet [136]. There is, however, limited information about effects of treating co-products from cereal grains with NaOH and it is also not known if oilseed meals other than SBM may benefit from treatment with NaOH. There is, therefore, a need for additional research in this area.

### Ammonia

Anhydrous ammonia, ammonium hydroxide, thermoammoniation, and urea also have been used to treat fibrous materials. A combination of ammonia and high pressure may improve solubilization and fermentability of fiber if fed to ruminants [11, 20], because this treatment may result in hydrolyzing the hemicellulose and cellulose fractions of the cell wall [12, 20], which make the cell wall more susceptible to be fermented by microbes [137]. Aqueous ammonia has also been used to remove the negative effects of aflatoxins B_1_ in corn fed to pigs [138]. It is possible that ammonia treatment may be used to increase the energy value of fibrous ingredients fed to pigs, but to our knowledge no research has been reported to test this hypothesis. However, if microbial fermentation of hemicellulose and cellulose is improved in ruminants fed ingredients that have been treated with ammonia, then it is likely that this may also be the case in pigs. There is, therefore, a need for conducting research to address this question and it may be prudent to conduct such research in gestating sows where transit time is slower than in growing pigs.

### Calcium oxide and calcium hydroxide

Calcium oxide or Ca(OH)_2_ also may be used to treat fibrous materials, but it is a less common treatment. However, castor seed meal treated with CaO may replace up to 330 g/kg of SBM in diets for dairy cows without affecting milk production or growth performance [13]. An experiment conducted by Lesoing et al. [14] demonstrated that digestibility of DM, OM, cellulose, and hemicelluloses increased in lambs fed wheat straw treated with Ca(OH)_2_ in combination with NaOH compared with lambs fed a diet with untreated wheat straw. Most of the research with CaO and Ca(OH)_2_ has been conducted with ruminant animals [139, 140], but it is not always these treatments resulted in improved growth performance of animals [141]. Treatment of fibrous materials with either CaO, Ca(OH)_2_, or NaOH solubilize more of the hemicellulose fraction than the cellulose fraction of the cell wall [14]. Calcium hydroxide also has been used to decontaminate corn infected with *Fusarium* mycotoxins [142], but data to demonstrate effects in non-ruminant animals are lacking.

## Enzyme treatments

Exogenous enzymes are commonly used in Northern European pig diets because most diets in Northern Europe are based on barley or wheat instead of corn. These ingredients have high concentrations of β-glucans and arabinoxylans [26, 143], and exogenous β-glucanases and xylanases may contribute to the hydrolysis of these fractions [144].

The effect of dietary exogenous carbohydrate digesting enzymes (hemicellulases, cellulases, xylanases, pectinases, β-glucanases, and α-galactosidases) on digestibility of energy and nutrients in corn and wheat DDGS fed to pig has been studied [21, 23], but results have been inconsistent. Pigs fed a barley-SBM diet supplemented with β-glucanases had increased energy and CP digestibility, but this was not the case if pigs were fed wheat-SBM, corn-SBM, or rye-SBM diets with addition of β-glucanases [145]. However, pigs that were fed a wheat-DDGS based diet that was supplemented with carbohydrase enzymes (xylanase, β-glucanase, and cellulase) had a greater GE digestibility compared with pigs fed diets that were not supplemented with enzymes [21]. In contrast, when xylanase was added to a corn-DDGS based diet, no improvement in energy digestibility was observed [23]. However, addition of xylanase to full fat or defatted rice bran resulted in a significant improvement in DE and ME of the ingredients, but that was not the case if xylanase was added to brewers rice, presumably due to a lack of substrate in brewers rice [24]. Addition of cellulase to DDGS may theoretically result in release of glucose that may be absorbed in the small intestine [20], but data to demonstrate this effect under practical conditions are lacking. Pigs fed a sorghum-SBM diet supplemented with the cellulase enzyme did not have improved growth performance or digestibility of DM, N, or GE compared with pigs fed a non-supplemented diet [19, 146]. Kim et al. [147] reported that pigs fed a corn-SBM based diet with addition of a cocktail of enzymes that contained α-galactosidase, β-mannanase, and β-mannosidase had improved energy and AA digestibility and improved G:F compared with pigs fed the same diet without enzymes. A similar response was reported by Omogbenigun et al. [148] who demonstrated that addition of enzymes (i.e., cellulase, galactanase, mannanase, and pectidase) as a cocktail had a positive effect on GE, starch, non-starch polysaccharides, and CP digestibility in diets containing corn, SBM, canola meal, barley, peas, wheat, and wheat by-products fed to pigs.

Exogenous enzymes usually are added during the diet mixing process. Therefore, these enzymes need to be thermostable if any thermal treatment is used. The enzymes also need to be stable in the conditions of the gastro intestinal tract of the pig to avoid reducing activity. However, if exogenous enzymes are used to treat ingredients before they are included in the diet, less variables need to be considered (e.g., thermal treatment, stomach pH, and time). Pigs fed a diet containing pretreated SBM with protease enzyme had no change in G:F compared with pigs fed the untreated SBM [149]. This is likely a result of the fact that no improvement in CP and AA digestibility is observed in protease-treated SBM compared with untreated SBM [150].

## Conclusions and Perspectives

Physical treatments available to treat feed ingredients and diets include roller and hammer mills and both of these types of mills may be used to grind feed for pigs, but there is a lack of knowledge about the ideal particle size that provides the best utilization of energy and nutrients. Newer grinding technologies include multistage grinding and stacked roller mills, but only limited information about the advantages of these technologies is available, but it is likely that as research becomes available, these technologies will gain increased popularity due to the more consistent particle size that can be produced. Thermal treatments that may be used include pelleting, extrusion, and expansion and positive effects of using all of these technologies have been well documented. It is, however, possible that combinations of physical and thermal treatments may be used to further improve the nutrient value of diets. There is, therefore, a need for research that addresses interactions among these technologies. It is also possible that some of the technologies are more appropriate in diets used in liquid feeding than in dry feeding, but no research has addressed this possibility.

Chemical treatments such as NaOH, ammonia, CaO, and Ca(OH)_2_ may also be used to improve the nutrient value of feed ingredients and diets, but very limited information about effects of these treatments on the nutritional value of pig diets have been reported. Carbohydrate digesting enzymes may be used individually or as cocktails to improve fermentation of the indigestible fractions of the diets, but the circumstances under which consistent positive results of use of carbohydrases in diets fed to pigs are obtained still have to be identified. Likewise, interactions among use of enzymes and diet processing technologies such as thermal or physical treatments have not been investigated and needs to be addressed in the future.

## Abbreviations

AA: Amino acids
ATTD: Apparent total tract digestibility
CP: Crude protein
DDGS: Distillers dried grain with solubles
DM: Dry matter
GE: gross energy
G:F: Gain to feed
ME: Metabolizable energy
NDF: Neutral detergent fiber
OM: Organic matter
SBM: Soybean meal
SID: Standardized ileal digestibility

## References

[1] Wondra KJ, Hancock JD, Behnke KC, Hines RH, Stark CR (1995). Effects of particle size and pelleting on growth performance, nutrient digestibility, and stomach morphology in finishing pigs. J Anim Sci.

[2] Herkelman KL, Cromwell GL, Stahly TS, Pfeiffer TW, Knabe DA (1992). Apparent digestibility of amino-acids in raw and heated conventional and low-trypsin-inhibitor soybeans for pigs. J Anim Sci.

[3] Lundblad KK, Issa S, Hancock JD, Behnke KC, McKinney LJ, Alavi S (2011). Effects of steam conditioning at low and high temperature, expander conditioning and extruder processing prior to pelleting on growth performance and nutrient digestibility in nursery pigs and broiler chickens. Anim Feed Sci Technol.

[4] Thomas M, van Zuilichem DJ, van der Poel AFB (1997). Physical quality of pelleted animal feed. 2. Contribution of processes and its conditions. Anim Feed Sci Technol.

[5] Hancock JD, Behnke KC, Lewis AJ, Southern LL (2001). Use of ingredient and diet processing technologies (grinding, mixing, pelleting, and extruding) to produce quality feeds for pigs. Swine Nutrition.

[6] Le Gall M, Warpechowski M, Jaguelin-Peyraud Y, Noblet J (2009). Influence of dietary fibre level and pelleting on the digestibility of energy and nutrients in growing pigs and adult sows. Animal.

[7] Stein HH, Bohlke RA (2007). The effects of thermal treatment of field peas (*Pisum sativum* L.) on nutrient and energy digestibility of growing pigs. J Anim Sci.

[8] Fahey GC, Bourquin LD, Titgemeyer EC, Atwell DG, Jung HG, Buxton DR, Hatfield DR, Ralph J (1993). Postharvest treatment of fibrous feedstuffs to improve their nutritive-value. Forage Cell Wall Structure and Digestibility.

[9] Felix TL, Murphy TA, Loerch SC (2012). Effects of dietary inclusion and NaOH treatment of dried distillers grains with solubles on ruminal metabolism of feedlot cattle. J Anim Sci.

[10] Morrow LA, Felix TL, Fluharty FL, Daniels KM, Loerch SC (2013). Effects of sulfur and acidity on performance and digestibility in feedlot lambs fed dried distillers grains with solubles. J Anim Sci.

[11] Realff MJ, Abbas C (2003). Industrial symbiosis. Refining the biorefinery. J Ind Ecol.

[12] Mosier N, Wyman C, Dale B, Elander R, Lee YY, Holtzapple M (2005). Features of promising technologies for pretreatment of lignocellulosic biomass. Bioresour Technol.

[13] Cobianchi JV, de Oliveira AS, Campos JMD, Guimaraes AV, Valadares SD, Cobianchi FP (2012). Productive performance and efficiency of utilization of the diet components in dairy cows fed castor meal treated with calcium oxide. Rev Bras Zootecn.

[14] Lesoing G, Klopfenstein T, Rush I, Ward J (1980). Chemical Treatment of Wheat Straw. J Anim Sci.

[15] Almeida FN, Stein HH (2010). Performance and phosphorus balance of pigs fed diets formulated on the basis of values for standardized total tract digestibility of phosphorus. J Anim Sci.

[16] Almeida FN, Sulabo RC, Stein HH (2013). Effects of a novel bacterial phytase expressed in aspergillus oryzae on digestibility of calcium and phosphorus in diets fed to weanling or growing pigs. J Anim Sci Biotechnol.

[17] González-Vega JC, Walk CL, Liu Y, Stein HH (2013). Endogenous intestinal losses of calcium and true total tract digestibility of calcium in canola meal fed to growing pigs. J Anim Sci.

[18] González-Vega JC, Walk CL, Stein HH (2015). Effects of microbial phytase on apparent and standardized total tract digestibility of calcium in calcium supplements fed to growing pigs. J Anim Sci.

[19] Park JS, Kim IH, Hancock JD, Hines RH, Cobb C, Cao H (2003). Effects of amylase and cellulase supplementation in sorghum-based diets for finishing pigs. Asian Australas J Anim Sci.

[20] Bals B, Dale B, Balan V (2006). Enzymatic hydrolysis of distiller’s dry grain and solubles (DDGS) using ammonia fiber expansion pretreatment. Energy Fuels.

[21] Emiola IA, Opapeju FO, Slominski BA, Nyachoti CM (2009). Growth performance and nutrient digestibility in pigs fed wheat distillers dried grains with solubles-based diets supplemented with a multicarbohydrase enzyme. J Anim Sci.

[22] Adeola O, Cowieson AJ (2011). Board-invited review: Opportunities and challenges in using exogenous enzymes to improve nonruminant animal production. J Anim Sci.

[23] Yañez JL, Beltranena E, Cervantes M, Zijlstra RT (2011). Effect of phytase and xylanase supplementation or particle size on nutrient digestibility of diets containing distillers dried grains with solubles cofermented from wheat and corn in ileal-cannulated grower pigs. J Anim Sci.

[24] Casas GA, Stein HH (2016). Effects of microbial xylanase on digestibility of dry matter, organic matter, neutral detergent fiber, and energy and the concentrations of digestible and metabolizable energy in rice coproducts fed to weanling pigs. J Anim Sci.

[25] Wondra KJ, Hancock JD, Kennedy GA, Behnke KC, Wondra KR (1995). Effects of reducing particle size of corn in lactation diets on energy and nitrogen metabolism in second-parity sows. J Anim Sci.

[26] Mavromichalis I, Hancock JD, Senne BW, Gugle TL, Kennedy GA, Hines RH (2000). Enzyme supplementation and particle size of wheat in diets for nursery and finishing pigs. J Anim Sci.

[27] Kim IH, Hancock JD, Hong JW, Cabrera MR, Hines RH, Behnke KC (2002). Corn particle size affects nutritional value of simple and complex diets for nursery pigs and broiler chicks. Asian Australas J Anim Sci.

[28] Fastinger ND, Mahan DC (2003). Effect of soybean meal particle size on amino acid and energy digestibility in grower-finisher swine. J Anim Sci.

[29] Rojas OJ, Stein HH (2015). Effects of reducing the particle size of corn grain on the concentration of digestible and metabolizable energy and on the digestibility of energy and nutrients in corn grain fed to growing pigs. Livest Sci.

[30] ASAE. Method of determining and expressing fineness of feed materials by sieving. Standard S319.4. American Society of Agricultural and Biological Engineers. MI: St. Joseph; 2008.

[31] Wondra KJ, Hancock JD, Behnke KC, Stark CR (1995). Effects of mill type and particle-size uniformity on growth performance, nutrient digestibility, and stomach morphology in finishing pigs. J Anim Sci.

[32] McEllhiney RR (1983). Roller mill grinding. Feed Management.

[33] Vermeer ME (1993). Roller mills versus hammermills: Grinding economics. Feed Manage.

[34] Reece FN, Lott BD, Deaton JW (1985). The effects of feed form, grinding method, energy-level, and gender on broiler performance in a moderate (21-C) environment. Poult Sci.

[35] Stark CS (2013). Particle size reduction, hammer mill and roller mill. Proc. 2013 Feed Manufacturing course.

[36] Healy BJ, Hancock JD, Kennedy GA, Bramelcox PJ, Behnke KC, Hines RH (1994). Optimum particle-size of corn and hard and soft sorghum for nursery pigs. J Anim Sci.

[37] Amaral NO, Amaral LGM, Cantarelli VS, Fialho ET, Zangeronimo MG, Rodrigues PB (2015). Influence of maize particle size on the kinetics of starch digestion in the small intestine of growing pigs. Anim Prod Sci.

[38] Wondra KJ, Hancock JD, Kennedy GA, Hines RH, Behnke KC (1995). Reducing particle size of corn in lactation diets from 1,200 to 400 micrometers improves sow and litter performance. J Anim Sci.

[39] Lawrence KR, Hastad CW, Goodband RD, Tokach MD, Dritz SS, Nelssen JL (2003). Effects of soybean meal particle size on growth performance of nursery pigs. J Anim Sci.

[40] Kim JC, Mullan BP, Pluske JR (2005). A comparison of waxy versus non-waxy wheats in diets for weaner pigs: Effects of particle size, enzyme supplementation, and collection day on total tract apparent digestibility and pig performance. Anim Feed Sci Technol.

[41] Oryschak MA, Simmins PH, Zijlstra RT (2002). Effect of dietary particle size and carbohydrase and/or phytase supplementation on nitrogen and phosphorus excretion of grower pigs. Can J Anim Sci.

[42] Kim JC, Mullan BP, Heo JM, Hansen CF, Pluske JR (2009). Decreasing dietary particle size of lupins increases apparent ileal amino acid digestibility and alters fermentation characteristics in the gastrointestinal tract of pigs. Br J Nutr.

[43] Montoya CA, Leterme P (2011). Effect of particle size on the digestible energy content of field pea (*Pisum sativum* L.) in growing pigs. Anim Feed Sci Technol.

[44] Liu P, Souza LWO, Baidoo SK, Shurson GC (2012). Impact of distillers dried grains with solubles particle size on nutrient digestibility, de and me content, and flowability in diets for growing pigs. J Anim Sci.

[45] Ton Nu MA, Blaabjerg K, Poulsen HD (2014). Effect of particle size and microbial phytase on phytate degradation in incubated maize and soybean meal. Animal.

[46] Giesemann MA, Lewis AJ, Hancock JD, Peo ERJ (1990). Effect of particle size of corn and grain sorghum on growth and digestibility by growing pigs. J Anim Sci.

[47] Healy BJ (1992). Nutritional value of selected sorghum grain for swine and poultry and effect of particle size on performance and intestinal morphology in young pigs and broiler chicks.

[48] Huang C, Zang JJ, Song PX, Fan PX, Chen JS, Liu DW (2015). Effects of particle size and drying methods of corn on growth performance, digestibility and haematological and immunological characteristics of weaned piglets. Arch Anim Nutr.

[49] Medel P, Garcia M, Lazaro R, de Blas C, Mateos GG (2000). Particle size and heat treatment of barley in diets for early-weaned piglets. Anim Feed Sci Technol.

[50] Yen JT. Anatomy of the digestive system and nutritional physiology. In: Swine Nutrition, Second Ed. Editted by Lewis AJ, Southern LL. Boca Raton: CRC Press; 2001;31–63.

[51] Mahan DC, Pickett RA, Perry TW, Curtin TM, Featherston WR, Beeson WM (1966). Influence of various nutritional factors and physical form of feed on esophagogastric ulcers in swine. J Anim Sci.

[52] Reimann EM, Maxwell CV, Kowalczyk T, Benevenga NJ, Grummer RH, Hoekstra WG (1968). Effect of fineness of grind of corn on gastric lesions and contents of swine. J Anim Sci.

[53] Pickett RA, Fugate WH, Harrington RB, Perry TW, Curtin TM (1969). Influence of feed preparation and number of pigs per pen on performance and occurrence of esophagogastric ulcers in swine. J Anim Sci.

[54] Maxwell CV, Reimann EM, Hoekstra WG, Kowalczyk T, Benevenga NJ, Grummer RH (1970). Effect of dietary particle size on lesion development and on contents of various regions of swine stomach. J Anim Sci.

[55] Ohara S, Ishihara K, Hotta K (1993). Regional differences in pig gastric mucins. Comp Biochem Physiol B-Biochem Mol Biol.

[56] Varum FJO, Veiga F, Sousa JS, Basit AW (2010). An investigation into the role of mucus thickness on mucoadhesion in the gastrointestinal tract of pig. Eur J Pharm Sci.

[57] Kowalczyk T (1969). Etiologic factors of gastric ulcers in swine. Am J Vet Res.

[58] Ramis G, Gomez S, Pallares FJ, Munoz A (2004). Influence of farm size on the prevalence of oesophagogastric lesions in pigs at slaughter in south-east spain. Vet Rec.

[59] Amory JR, Mackenzie AM, Pearce GP (2006). Factors in the housing environment of finisher pigs associated with the development of gastric ulcers. Vet Rec.

[60] Friendship RA. Gastric ulcers: An under-recognized cause of mortality and morbidity. In: Advances in Pork Production. Banff: 2003.

[61] Swaby H, Gregory NG (2012). A note on the frequency of gastric ulcers detected during post-mortem examination at a pig abattoir. Meat Sci.

[62] Rodriguez BD, Taborda DA, Ortiz LC (2009). Association of gastric ulcer and helicobacter spp. In pigs in antioquia, colombia. Rev Colomb Cienc Pec.

[63] Mößeler A, Köttendorf S, Liesner VG, Kamphues J (2010). Impact of diets’ physical form (particle size; meal/pelleted) on the stomach content (dry matter content, ph, chloride concentration) of pigs. Livest Sci.

[64] Morgan DR, Fox JG, Leunk RD (1991). Comparison of isolates of helicobacter-pylori and helicobacter-mustelae. J Clin Microbiol.

[65] Eaton KA, Radin MJ, Krakowka S (1995). An animal-model of gastric-ulcer due to bacterial gastritis in mice. Vet Pathol.

[66] Rojas OJ, Liu Y, Stein HH (2016). Effects of particle size of yellow dent corn on physical characteristics of diets and growth performance and carcass characteristics of growing-finishing pigs. J Anim Sci.

[67] Seerley RW, Vandergrift WL, Hale OM (1988). Effect of particle-size of wheat on performance of nursery, growing and finishing pigs. J Anim Sci.

[68] Paulk CB, Hancock JD, Fahrenholz AC, Wilson JM, Mckinny LJ, Behnke KC (2015). Effects of sorghum particle size on milling characteristics and growth performance in finishing pigs. Anim Feed Sci Technol.

[69] Appel WB, McElhiney RR (1994). Physical properties of feed ingredients. Feed Manufacturing Technology.

[70] Skoch ER, Binder SF, Deyoe CW, Allee GL, Behnke KC (1983). Effects of pelleting conditions on performance of pigs fed a corn-soybean meal diet. J Anim Sci.

[71] Pettigrew JE, Miller KP, Moser RL, Cornelius SG. Feed intake of lactating sows as affected by fineness of corn grind. In: Minnesota Swine Research. Report. No. AG-BU-2300. Department of Animal Science, University of Minnesota. MN: St. Paul. 1985. p. 54–5.

[72] Ohh SH, Han KN, Chae BJ, Han IK, Acda SP (2002). Effects of feed processing methods on growth performance and ileal digestibility of amino acids in young pigs. Asian Australas J Anim Sci.

[73] Rehman ZU, Shah WH (2005). Thermal heat processing effects on antinutrients, protein and starch digestibility of food legumes. Food Chem.

[74] Jha R, Overend DN, Simmins PN, Hickling D, Zijlstra RT (2011). Chemical characteristics, feed processing quality, growth performance and energy digestibility among wheat classes in pelleted diets fed to weaned pigs. Anim Feed Sci Technol.

[75] Liu SY, Selle PH, Cowieson AJ (2013). Strategies to enhance the performance of pigs and poultry on sorghum-based diets. Anim Feed Sci Technol.

[76] Rojas OJ, Vinyeta E, Stein HH (2016). Effects of pelleting, extrusion, or extrusion and pelleting on energy and nutrient digestibility in diets containing different levels of fiber and fed to growing pigs. J Anim Sci.

[77] Zijlstra RT, Tibble S, van Kempen TATG, Torrallardona D, Roura E (2009). Feed manufacturing technology and feed intake in young pigs. Voluntary Feed Intake in Pigs.

[78] Gerrard JA (2002). New aspects of an ageing chemistry - recent developments concerning the Maillard reaction. Aust J Chem.

[79] Nursten HE (2005). Nutritional aspects. The Maillard reaction: Chemistry, Biochemistry, and Implications.

[80] Fontaine J, Zimmer U, Moughan PJ, Rutherfurd SM (2007). Effect of heat damage in an autoclave on the reactive lysine contents of soy products and corn distillers dried grains with solubles. Use of the results to check on lysine damage in common qualities of these ingredients. J Agric Food Chem.

[81] González-Vega JC, Kim BG, Htoo JK, Lemme A, Stein HH (2011). Amino acid digestibility in heated soybean meal fed to growing pigs. J Anim Sci.

[82] Almeida FN, Htoo JK, Thomson J, Stein HH (2013). Amino acid digestibility of heat damaged distillers dried grains with solubles fed to pigs. J Anim Sci Biotechnol.

[83] Sauber TE, Owens FN (2000). Cereal grains and by-products for swine. Swine Nutrition.

[84] Slominski BA, Davie T, Nyachoti MC, Jones O (2007). Heat stability of endogenous and microbial phytase during feed pelleting. Livest Sci.

[85] Svihus B, Zimonja O (2011). Chemical alterations with nutritional consequences due to pelleting animal feeds: A review. Anim Prod Sci.

[86] Obernberger I, Thek G (2010). Pellet production and logistics. The pellet handbook- The Production and Thermal Utilisation of Pellets.

[87] Lewis LL, Stark CR, Fahrenholz AC, Bergstrom JR, Jones CK (2015). Evaluation of conditioning time and temperature on gelatinized starch and vitamin retention in a pelleted swine diet. J Anim Sci.

[88] Fellows P, Fellows P (2000). Extrusion. Food Processing Technology: Principles and Practice.

[89] Brown IL (2004). Applications and uses of resistant starch. J AOAC Int.

[90] Htoon A, Shrestha AK, Flanagan BM, Lopez-Rubio A, Bird AR, Gilbert EP (2009). Effects of processing high amylose maize starches under controlled conditions on structural organisation and amylase digestibility. Carbohydr Polym.

[91] Lahaye L, Riou Y, Seve B (2007). The effect of grinding and pelleting of wheat and maize on amino acids true ileal digestibility and endogenous losses in growing pigs. Livest Sci.

[92] Thomas M, van der Poel AFB (1996). Physical quality of pelleted animal feed. 1. Criteria for pellet quality. Anim Feed Sci Technol.

[93] Traylor SL, Behnke KC, Hancock JD, Hines RH, Johnston SL, Chae BJ (1999). Effects of expander operating conditions on nutrient digestibility in finishing pigs. Asian Australas J Anim Sci.

[94] Steidinger MU, Goodband RD, Tokach MD, Dritz SS, Nelssen JL, McKinney LJ (2000). Effects of pelleting and pellet conditioning temperatures on weanling pig performance. J Anim Sci.

[95] Jensen AH, Becker DE (1965). Effect of pelleting diets and dietary components on performance of young pigs. J Anim Sci.

[96] Lahaye L, Ganier P, Thibault JN, Riou Y, Seve B (2008). Impact of wheat grinding and pelleting in a wheat-rapeseed meal diet on amino acid ileal digestibility and endogenous losses in pigs. Anim Feed Sci Technol.

[97] Ginste JV, de Schrijver R (1998). Expansion and pelleting of starter, grower and finisher diets for pigs: Effects on nitrogen retention, ileal and total tract digestibility of protein, phosphorus and calcium and in vitro protein quality. Anim Feed Sci Technol.

[98] Skoch ER, Binder SF, Deyoe CW, Allee GL, Behnke KC (1983). Effects of steam pelleting conditions and extrusion cooking on a swine diet containing wheat middlings. J Anim Sci.

[99] Noblet J, van Milgen J (2004). Energy value of pig feeds: effect of pig body weight and energy evaluation system. J Anim Sci.

[100] Kim BG, Kil DY, Stein HH (2013). In growing pigs, the true ileal and total tract digestibility of acid hydrolyzed ether extract in extracted corn oil is greater than in intact sources of corn oil or soybean oil. J Anim Sci.

[101] Kil DY, Sauber TE, Jones DB, Stein HH (2010). Effect of the form of dietary fat and the concentration of dietary neutral detergent fiber on ileal and total tract endogenous losses and apparent and true digestibility of fat by growing pigs. J Anim Sci.

[102] Rojas OJ (2015). Use of feed technology to improve the nutritional value of feed ingredients and diets fed to pigs.

[103] Walker N (1989). A comparison of wheat-based or barley-based diets given ad-libitum as meal or pellets to finishing pigs. Anim Feed Sci Technol.

[104] Xing JJ, van Heugten E, Li DF, Touchette KJ, Coalson JA, Odgaard RL (2004). Effects of emulsification, fat encapsulation, and pelleting on weanling pig performance and nutrient digestibility. J Anim Sci.

[105] Lewis LL, Stark CR, Fahrenholz AC, Goncalves MAD, DeRouchey JM, Jones CK (2015). Effects of pelleting conditioner retention time on nursery pig growth performance. J Anim Sci.

[106] Paulk CB, Hancock JD (2016). Effects of an abrupt change between diet form on growth performance of finishing pigs. Anim Feed Sci Technol.

[107] Laitat M, Vandenheede M, Desiron A, Canart B, Nicks B (1999). Comparison of performance, water intake and feeding behaviour of weaned pigs given either pellets or meal. Anim Sci.

[108] Overholt MF, Lowell JE, Arkfeld EK, Grossman IM, Stein HH, Dilger AC (2016). Effects of pelleting diets without or with distillers’ dried grains with solubles on growth performance, carcass characteristics, and gastrointestinal weights of growing-finishing barrows and gilts. J Anim Sci.

[109] Ulens T, Demeyer P, Ampe B, Van Langenhove H, Millet S (2015). Effect of grinding intensity and pelleting of the diet on indoor particulate matter concentrations and growth performance of weanling pigs. J Anim Sci.

[110] NRC (2012). Nutrient requirements of swine.

[111] Richert BT, DeRouchey JM (2010). Swine feed processing and manufacturing. National Swine Nutrition Guide.

[112] Fry RS, Hu W, Williams SB, Paton ND, Cook DR (2012). Diet form and by-product level affect growth performance and carcass characteristics of grow-finish pigs. J Anim Sci.

[113] Spears JK, Fahey GC (2004). Resistant starch as related to companion animal nutrition. J AOAC Int.

[114] Sauer WC, Mosenthin R, Pierce AB (1990). The utilization of pelleted, extruded, and extruded and repelleted diets by early weaned pigs. Anim Feed Sci Technol.

[115] Durmic Z, Pethick DW, Mullan BP, Accioly JM, Schulze H, Hampson DJ (2002). Evaluation of large-intestinal parameters associated with dietary treatments designed to reduce the occurrence of swine dysentery. Br J Nutr.

[116] Muley NS, van Heugten E, Moeser AJ, Rausch KD, van Kempen TATG (2007). Nutritional value for swine of extruded corn and corn fractions obtained after dry milling. J Anim Sci.

[117] Chae BJ, Han IK, Kim JH, Yang CJ, Chung YK, Rhee YC (1997). Effects of extrusion conditions of corn and soybean meal on the physico-chemical properties, ileal digestibility and growth of weaned pig. Asian Australas J Anim Sci.

[118] Navarro DMDL, Liu Y, Bruun TS, Stein HH (2014). Amino acid digestibility in processed soybean products and rapeseed products fed to weanling pigs. J Anim Sci.

[119] Htoo JK, Meng X, Patience JF, Dugan MER, Zijlstra RT (2008). Effects of coextrusion of flaxseed and field pea on the digestibility of energy, ether extract, fatty acids, protein, and amino acids in grower-finisher pigs. J Anim Sci.

[120] Urriola PE, Shurson GC, Stein HH (2010). Digestibility of dietary fiber in distillers coproducts fed to growing pigs. J Anim Sci.

[121] Fancher BI, Rollins D, Trimbee B (1996). Feed processing using the annular gap expander and its impact on poultry performance. J Appl Poult Res.

[122] van der Poel AFB, Schoterman A, Bosch MW (1998). Effect of expander conditioning and/or pelleting of a diet on the ileal digestibility of nutrients and on feed intake after choice feeding of pigs. J Sci Food Agric.

[123] Callan JJ, Garry BP, O’Doherty JV (2007). The effect of expander processing and screen size on nutrient digestibility, growth performance, selected faecal microbial populations and faecal volatile fatty acid concentrations in grower-finisher pigs. Anim Feed Sci Technol.

[124] Laurinen P, Valaja J, Nasi M, Smeds K (1998). Effects of different expander processing conditions on the nutritive value of barley and wheat by-products in pig diets. Anim Feed Sci Technol.

[125] Johnston SL, Hines RH, Hancock JD, Behnke KC, Traylor SL, Chae BJ (1999). Effects of expander conditioning of complex nursery diets on growth performance of weanling pigs. Asian Australas J Anim Sci.

[126] Lundblad KK, Hancock JD, Behnke KC, Prestlokken E, McKinney LJ, Sorensen M (2009). The effect of adding water into the mixer on pelleting efficiency and pellet quality in diets for finishing pigs without and with use of an expander. Anim Feed Sci Technol.

[127] Millet S, Kumar S, De Boever J, Ducatelle R, De Brabander D (2012). Effect of feed processing on growth performance and gastric mucosa integrity in pigs from weaning until slaughter. Anim Feed Sci Technol.

[128] Stein HH, Liu K, Rosentrater KA (2012). Feeding ethanol coproducts to swine. Distillers Grains: Production, Properties, and Utilization.

[129] Kim WK, Patterson PH (2000). Nutritional value of enzyme- or sodium hydroxide-treated feathers from dead hens. Poult Sci.

[130] Braman WL, Abe RK (1977). Laboratory and in vivo evaluation of nutritive-value of naoh-treated wheat straw. J Anim Sci.

[131] Hunt CW, Paterson JA, Zinn GM, Williams JE (1984). Effect of particle length and sodium-hydroxide treatment of wheat straw on site and extent of digestion by lambs. J Anim Sci.

[132] Miron J, BenGhedalia D, Solomon R (1997). Digestibility by dairy cows of monosaccharide components in diets containing either ground sorghum or sorghum grain treated with sodium hydroxide. J Dairy Sci.

[133] Kemm EH, Ras MN (1985). A comparison between sodium-hydroxide treated and untreated bird-proof sorghum in pig growth diets. South Afr J Anim Sci.

[134] Echeverria V, Belmar R, Ly J, Santos-Ricalde RH (2002). Effect of leucaena leucocephala leaf meal treated with acetic acid or sodium hydroxide on apparent digestibility and nitrogen retention in pig diets. Anim Feed Sci Technol.

[135] Acamovic T, D’Mello JPF, Colegate SM, Dorling PR (1994). Influence of *Leucaena* seed and leaf meal diets on young chicks. Plant-Associated Toxins: Agricultural, Phytochemical and Ecological Aspects.

[136] Young LG, Smith GC (1973). Processing soybeans with sodium-hydroxide and copper-sulfate for pigs. Can J Anim Sci.

[137] Oji UI, Etim HE, Okoye FC (2007). Effects of urea and aqueous ammonia treatment on the composition and nutritive value of maize residues. Small Ruminant Res.

[138] Jensen AH, Brekke OL, Frank GR, Peplinski AJ (1977). Acceptance and utilization by swine of aflatoxin-contaminated corn treated with aqueous or gaseous ammonia. J Anim Sci.

[139] Magalhaes FA, Filho SDV, Menezes GCD, Gionbelli MP, Zanetti D, Machado MG (2012). Intake and performance of feedlot cattle fed diets based on high and low brix sugar cane with or without calcium oxide and corn silage. Rev Bras Zootecn.

[140] Polyorach S, Wanapat M (2015). Improving the quality of rice straw by urea and calcium hydroxide on rumen ecology, microbial protein synthesis in beef cattle. J Anim Physiol Anim Nutr.

[141] Duckworth M (2013). Effects of feeding CaO treated modified wet distillers grains with solubles or corn stover to cattle on performance, carcass characteristics, and ruminal metabolism.

[142] Rempe I, Brezina U, Kersten S, Danicke S (2013). Effects of a fusarium toxin-contaminated maize treated with sodium metabisulphite, methylamine and calcium hydroxide in diets for female piglets. Arch Anim Nutr.

[143] Li S, Sauer WC, Huang SX, Gabert VM (1996). Effect of beta-glucanase supplementation to hulless barley- or wheat-soybean meal diets on the digestibilities of energy, protein, beta-glucans, and amino aids in young pigs. J Anim Sci.

[144] Owusu-Asiedu A, Simmins PH, Brufau J, Lizardo R, Peron A (2010). Effect of xylanase and beta-glucanase on growth performance and nutrient digestibility in piglets fed wheat-barley-based diets. Livest Sci.

[145] Li S, Sauer WC, Mosenthin R, Kerr B (1996). Effect of beta-glucanase supplementation of cereal-based diets for starter pigs on the apparent digestibilities of dry matter, crude protein and energy. Anim Feed Sci Technol.

[146] Kim IH, Hancock JD, Hines RH, Kim CS (1998). Effects of cellulase enzymes and bacterial feed additives on the nutritional value of sorghum grain for finishing pigs. Asian Australas J Anim Sci.

[147] Kim SW, Knabe DA, Hong KJ, Easter RA (2003). Use of carbohydrases in corn-soybean meal-based nursery diets. J Anim Sci.

[148] Omogbenigun FO, Nyachoti CM, Slominski BA (2004). Dietary supplementation with multienzyme preparations improves nutrient utilization and growth performance in weaned pigs. J Anim Sci.

[149] Rooke JA, Slessor M, Fraser H, Thomson JR (1998). Growth performance and gut function of piglets weaned at four weeks of age and fed protease-treated soya-bean meal. Anim Feed Sci Technol.

[150] Caine WR, Sauer WC, Tamminga S, Verstegen MWA, Schulze H (1997). Apparent ileal digestibilities of amino acids in newly weaned pigs fed diets with protease-treated soybean meal. J Anim Sci.

